# Supplementary material for: Trans-marine dispersal inferred from the saltwater tolerance of lizards from Taiwan
Source: PLoS One. 2021 Feb 12;16(2):e0247009. doi: 10.1371/journal.pone.0247009 (PMC7880474; doi:10.1371/journal.pone.0247009)
Supplement: S1 File — The Taiwan and adjacent islands with the coastline (A). The long dashed line represents the coastline during the Last Glacial Maximum 26.5–18 ka, and the short dashed line represents the coastline before 1.55 Ma. The green patterns represent the distribution of P. elegans (B), E. longicaudata (C), D. swinhonis (D), H. frenatus (E), A. sagrei (F) and E. multifasciata (G). The distribution of the species was referenced from The Atlas of Amphibians and Reptiles of Taiwan [68] and the Global Biodiversity Information Facility (http://www.gbif.org/). The coastlines were based on the open data from Natural Earth, which were licensed under the Public Domain. (ZIP) [file pone.0247009.s001.zip › Minhao Supplementary Information 20200601.docx]

Supplementary Information

Over-ocean dispersal inferred from the saltwater tolerance of lizards from Taiwan

Min-Hao Hsu^1,2^, Jhan-Wei Lin^1^, Chen-Pan Liao^1,3^, Jung-Ya Hsu^1^, Wen-San Huang^1,2,3*^

^1^Department of Biology, National Museum of Natural Science, Taichung, Taiwan

^2^Department of Life Sciences, National Chung Hsing University, Taichung, Taiwan

^3^Department of Life Science, Tunghai University, Taichung, Taiwan

*Corresponding author: [wshuang.380@gmail.com](mailto:wshuang.380@gmail.com)

**The distributions of six species in this study**

The four native species were *Plestiodon elegans*, existing throughout Taiwan, the western and northern islands, and south-eastern China (Fig. S1B); *Eutropis longicaudata*, distributed in southern Taiwan, the southern islands, and mainland Southeast Asia (Fig. S1C); *Diploderma swinhonis*, existing throughout Taiwan, the southern islands, and the Ryukyu Islands (Fig. S1D); and *Hemidactylus frenatus*, existing throughout Taiwan, all adjacent islands, and almost every island in East and Southeast Asia (Fig. S1E). The two introduced species were *Eutropis multifasciata* and *Anolis sagrei*. The former is native to the Philippines and was first discovered in southern Taiwan in 1992 ^1^. This species has been found in Green Island since 2008 ^2^ and in Orchid Island since 2017 (Fig. S1F). *A. sagrei* from the West Indies was discovered in central Taiwan in 2000 ^3^ and eastern Taiwan in 2006 ^4^ but has not been discovered to date in adjacent islands (Fig. S1G).


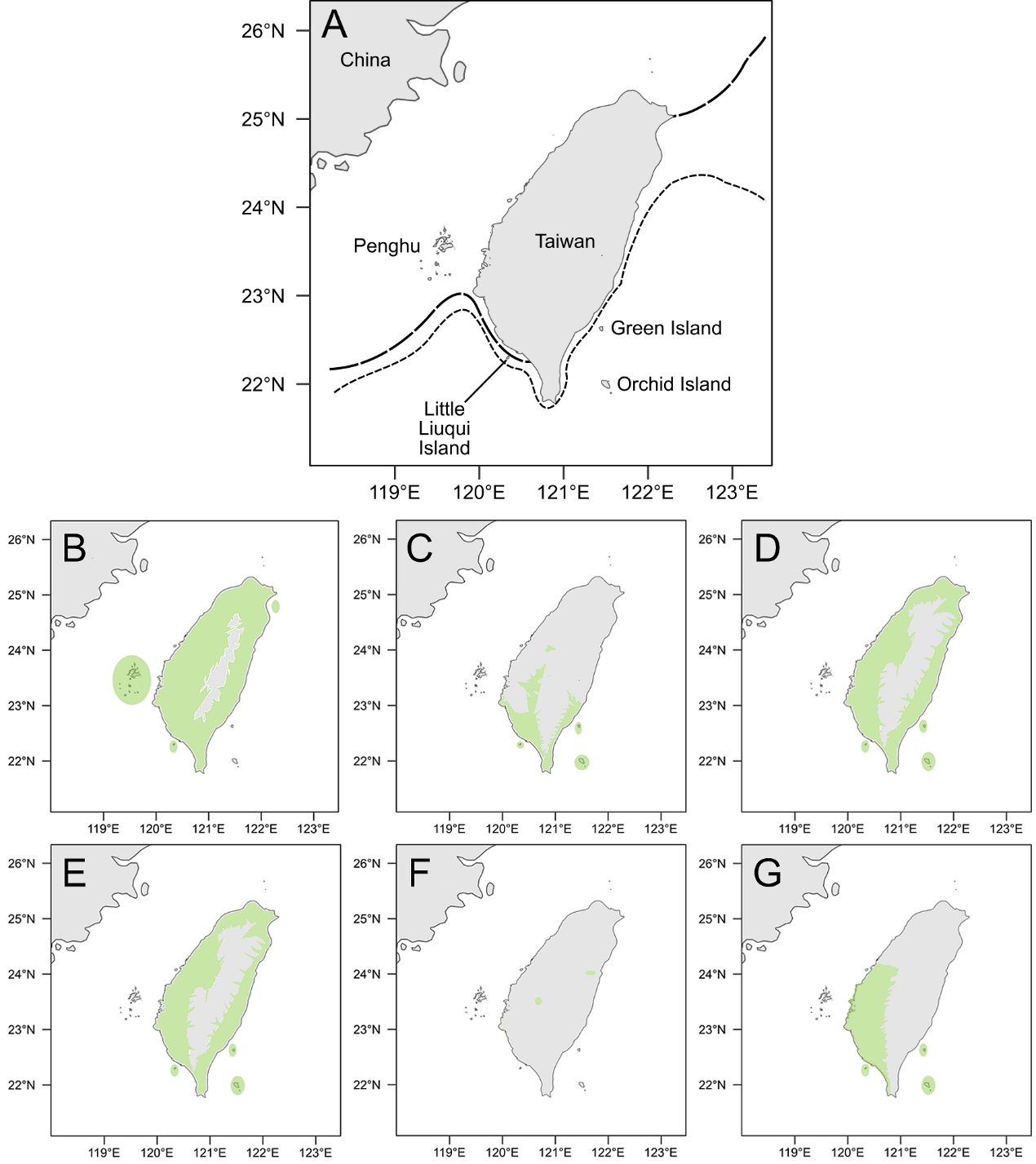


**S1 Fig. The historical land connection with the Asian continent in Taiwan and current distribution of the six lizard species.** The long dashed line represents the coastline during the Last Glacial Maximum 26.5–18 ka, and the short dashed line represents the coastline before 1.55 Ma (A). The green patterns represent the distribution of *P. elegans* (B), *E. longicaudata* (C), *D. swinhonis* (D), *H. frenatus* (E), *A. sagrei* (F) and *E. multifasciata* (G).

**References**

1 Ota, H., Chang, H. W., Liu, K. C. & Hikida, T. A new record of the viviparous skink, *Mabuya multifasciata* (Kuhl, 1820)(Squamata: Reptilia), from Taiwan. *Zool. Stud.* **33**, 86-89 (1994).

2 Chen, P.-C. *et al.* The Research of Terrestrial Vertebrate Fauna on the Green Island. *Marine National Park Headquarters, Kaohsiung, Taiwan.* (2008).

3 Norval, G., Mao, J. J., Chu, H. P. & Chen, L. C. A new record of an introduced species, the brown anole (*Anolis sagrei*)(Duméril & Bibron, 1837), in Taiwan. *Zool. Stud.* **41**, 332-336 (2002).

4 Chang, N. C. A new discovered alien lizard of Hualien—*Anolis sagrei*. *Nat. Conserv. Quarter.* **57**, 37-41 (2007).
