# Supplementary material for: Trans-marine dispersal inferred from the saltwater tolerance of lizards from Taiwan
Source: PLoS One. 2021 Feb 12;16(2):e0247009. doi: 10.1371/journal.pone.0247009 (PMC7880474; doi:10.1371/journal.pone.0247009)
Supplement: Attachment — Submitted filename: Response to Reviewers (4).docx [file pone.0247009.s002.docx]

Response to Reviewers

Response: We responded the additional requirements of the editor, the questions and the suggestions of the reviewer under each item.

Journal Requirements:

1. Please ensure that your manuscript meets PLOS ONE's style requirements, including those for file naming. The PLOS ONE style templates can be found at <https://journals.plos.org/plosone/s/file?id=wjVg/PLOSOne_formatting_sample_main_body.pdf> and

<https://journals.plos.org/plosone/s/file?id=ba62/PLOSOne_formatting_sample_title_authors_affiliations.pdf>

Response: We checked the manuscripts and files to meet PLOS ONE's style requirements.

2. In your Methods section, please provide additional information regarding the permits you obtained for the work. Please ensure you have included the full name of the authority that approved the field site access and, if no permits were required, a brief statement explaining why.

Response: We had added descriptions about the permits for collection, husbandry and treatment procedures (Line 175-178) and uploaded PLOS ONE human endpoints checklist in previous resubmission. The full name of the authority that approved the field site access was checked again in this version.

3. We note that Figure(s) 4 and S1 in your submission contain map images which may be copyrighted. All PLOS content is published under the Creative Commons Attribution License (CC BY 4.0), which means that the manuscript, images, and Supporting Information files will be freely available online, and any third party is permitted to access, download, copy, distribute, and use these materials in any way, even commercially, with proper attribution. For these reasons, we cannot publish previously copyrighted maps or satellite images created using proprietary data, such as Google software (Google Maps, Street View, and Earth). For more information, see our copyright guidelines: <http://journals.plos.org/plosone/s/licenses-and-copyright>.

Response: In both Figure 4 and Supplementary S1 Fig, the coastlines were based on the open data from Natural Earth and Taiwan’s Ministry of the Interior, which were licensed under the Public Domain and the Open Government Data License (version 1.0; <https://data.gov.tw/en/license>), respectively.

4. Please include captions for your Supporting Information files at the end of your manuscript, and update any in-text citations to match accordingly. Please see our Supporting Information guidelines for more information: http://journals.plos.org/plosone/s/supporting-information.

Response: We added the caption of Supporting Information files at the end of the manuscript as the requirement.

5. Review Comments to the Author

Response: We responded the questions and the suggestions of the reviewer under each item. We also added the annotation that included the suggestions of the reviewer and our responses.

Reviewer #1: The ms on dispersal inferred from the saltwater tolerance of lizards from Taiwan, is an interesting work worth publishing in PLOS ONE after revision of the points mentioned below.

Line 1: consider “trans-marine” instead of “over-ocean”

Response: Revised as suggested.

Line 50: “many introduced” change to “many species now recorded as introduced” or something similar

Response: Revised as suggested.

Line 52: delete “artificial”

Response: Revised as suggested.

Line 92: raft or artificial boats > rafts or boats

Response: Revised as suggested.

Line 92: Consider changing “In” with “Regarding” or similar

Response: Revised as suggested.

Line 106-107: I suggest rephrasing to “Taiwan and the adjacent islands are separated from the Asian continent by a sea strait with a depth of approximately 70 metres”. Additionally, “metres” is UK English style. If the journal requires US English you should change to “meters”.

Response: We rephrased the sentence and changed the words of the UK English to US English as suggested.

Lines 135-143: Table 1. Please add a symbol (e.g. an asterisk) next to the name of the introduced species to facilitate readers not familiar with the local fauna. Adjust the legend accordingly.

Response: We added the asterisks before the name of the introduced species in Table 1, and added the description at the end of the legend.

Line 158: failed to lay eggs

Response: Revised as suggested.

Line 164: any individual observed having (oral?) secretions

Response: Revised as suggested.

Line 172: times

Response: Revised as suggested.

Line 179: in > on

Response: Revised as suggested.

Line 180: that individual directly contact with seawater. Please rephrase

Response: We rephrased the sentence as suggested.

Line 185-186: “The water surface slightly exceeded the abdomen of the lizard but did not exceed the mouth”. How did you manage this? Did you have small rafts, or by controlling the water level in the tank to have it shallow enough for the animal’s feet to touch the bottom? Please clarify.

Response: We added the description as suggested.

Line 192: Are there published data on the selected temperatures Tset (Hertz et al 1993)? It would be interesting as if e.g. species A has a preferred T=36oC it would maybe react differently than species B with a preferred temperature of 31oC

Response: We choose this temperature because we found those species active well from 25-29 C in the wild. That’s why we use this specific temperature. We indeed do not have any data which species prefer in which temperature.

Line 196: Either 6 or 7.

Response: Revised as suggested.

Line 199: delete “in this study”

Response: Revised as suggested.

Lines 199-200: the incubation > their respective incubation

Response: Revised as suggested.

Line 208: delete “quite”

Response: Revised as suggested.

Lines 233-235: Notably…treatment. The phrase as is, is difficult to understand. Please rephrase

Response: We revised to make it easier to understand.

Lines 269-271: Not sure this is a correct approach. Most individuals of at least 2 species did survive. Why not include at least them in the comparisons? Moreover, even the weights of the dead specimen could be reported (maybe separately). Both info mentioned above would help understand if it was desiccation that caused deaths (i.e. severe weight loss) or another reason (e.g. possible intake of water orally causing salt water poisoning or other). You could at least add one or two lines commenting an this.

Response: Our experimental design was a factorial design, which lizard species, sex and salinity were three crossing factors. Because our results showed that the survival rates of four lizrad species were less than 30 %, to estimate those RTWLs were difficult, and it is impossible to include all SW results in the model duo to some completely empty cells. This is the reason we completely excluded the data of SW treatment in the model. However, we appended the RTWLs of the two survived species (*E. longicaudata* and *E. multifasciata*) in the same paragraph.

Line 336: …incubation rate. Do you mean hatching success?

Response: Revised as suggested.

Line 339: …hatchling success. Do you mean hatching success?

Response: Revised as suggested.

Line 346: arrive > arrived at

Response: Revised as suggested.

Line 348: … then connect this. Consider changing to “and possible connections of their physiological traits to their dispersal abilities” or something similar

Response: Revised as suggested.

Line 354: …if it could drift to Green Island and Orchid Island. I think this has no meaning, at least I do not understand it. Better omit or otherwise explain.

Response: We deleted the description to avoid the possibility of misunderstanding.

Lines 365-366: …that they – native. The phrase as is, is difficult to understand. Please rephrase

Response: Revised as suggested.

Line 341: Discussion. The entire discussion could be split in two sub paragraphs. The first till line 400 where you discuss the actual experiment’s results and the rest i.e. from line 401 onward where you discuss biogeographic scenarios.

Response: We splitted discussion into two subsection by inserting two subsection header lines.

Line 379: …than in the other species. Which other? the ones in the study? If yes, please specify

Response: We added the description in the sentence.

Line 418: drifting > oceanic drifts (actually, both “drifting” and “rafting” show energetic activity, whereas both are passive/involuntary. You could as well change “rafting” to “natural rafts”)

Response: Revised as suggested.

Line 423: distributes widely > have a wide distribution
Response: Revised as suggested.

Line 423: ocean > oceanic
Response: Revised as suggested.

Line 428: shortens the drifting > shorten the dispersal (or similar)
Response: Revised as suggested.

Line 449: is accordance > is in accordance
Response: Revised as suggested.

Line 451: disperses > disperse
Response: Revised as suggested.

Lines 451 - 453: Yes, but this does not exclude possible translocation by humans

Response: We added the description after the sentence as suggested.

Line 459: consider changing could to should

Response: Revised as suggested.

Line 463: delete “been”

Response: Revised as suggested.

Line 466: insert “the” before Kuroshio

Response: Revised as suggested.
